# Intra-Articular Slow-Release Triamcinolone Acetonide from Polyesteramide Microspheres as a Treatment for Osteoarthritis

**DOI:** 10.3390/pharmaceutics13030372

**Published:** 2021-03-11

**Authors:** Anna Tellegen, Martijn Beukers, Imke Rudnik-Jansen, Nicolien van Klaveren, Kan Loi How, Nina Woike, George Mihov, Jens Thies, Erik Teske, Laura Creemers, Marianna Tryfonidou, Björn Meij

**Affiliations:** 1 Department of Clinical Sciences, Faculty of Veterinary Medicine, Utrecht University, Yalelaan 108, 3584 CM Utrecht, The Netherlands; a.r.tellegen@uu.nl (A.T.); m.beukers@uu.nl (M.B.); e.teske@uu.nl (E.T.); b.p.meij@uu.nl (B.M.); 2 Department of Orthopaedics, University Medical Centre Utrecht, Heidelberglaan 100, 3584 CX Utrecht, The Netherlands; i.r@inano.au.dk (I.R.-J.); l.b.creemers@umcutrecht.nl (L.C.); 3 Orthopaedics Department, Medisch Centrum voor Dieren, Isolatorweg 45, 1014 AS Amsterdam, The Netherlands; njvanklaveren@hotmail.com; 4 Diergeneeskundig Specialisten Centrum Den Haag, Regentesselaan 190, 2562 EH Den Haag, The Netherlands; how@wxs.nl; 5 DSM Biomedical, Koestraat 1, 6167 RA Geleen, The Netherlands; nina.woike@dsm.com (N.W.); george.mihov@dsm.com (G.M.); jens.thies@dsm.com (J.T.)

**Keywords:** anti-inflammatory drugs, biomaterials, controlled release, corticosteroid, degenerative joint disease

## Abstract

Osteoarthritis (OA) is a common cause of pain and disability. Local corticosteroid injections are effective in treating OA pain and inflammation but are short-acting. Prolonged intra-articular (IA) corticosteroid exposure may even lead to cartilage deterioration. The aim of this prospective study was to assess safety and provide proof-of-concept of IA-applied biodegradable polyesteramide-based microspheres (PEAMs) gradually releasing triamcinolone acetonide (TA). Mimicking continuous exposure associated with local drug delivery in canine articular chondrocytes cultured in the continuous presence of TA tissue regeneration was not affected, whereas intermittent exposure reduced proteoglycan production. In this respect, TA-PEAMs administered IA in a proof-of-concept study in 12 client-owned dogs with established OA also showed safety by radiographic examination, without changes in OA severity and in glycosaminoglycan synovial fluid levels. Treatment also resulted in clinical improvement in 10 out of 11 dogs during the two-month follow-up period, which persisted in 6 out of 10 dogs after 6 months, based on objective gait analysis and owner questionnaires. Synovial prostaglandin E_2_, a pro-inflammatory marker, was decreased two months after treatment. This study showed safety and proof-of-concept of IA-administered TA-PEAMs in dogs with OA, as a first step towards translation into the veterinary and human clinic.

## 1. Introduction

Osteoarthritis (OA) is a debilitating and chronic disease, leading to loss of quality of life and decreased productivity, which in turn leads to increased socioeconomic costs. With the increase in life expectancy, its prevalence is expected to rise [1]. Pain, swelling and stiffness of the affected joint are the main clinical signs of OA [2]. OA is characterized by degeneration of articular cartilage, accompanied by pathological changes in subchondral bone and the synovial lining, such as osteophyte formation and synovial inflammation. Synovial inflammation, among others mediated by the pro-inflammatory cytokine prostaglandin E_2_ (PGE_2_), was found to be responsible for both OA symptoms and progression [2,3]. Oral (non-steroidal) anti-inflammatory drugs and other analgesics are frequently used to treat OA-related pain and can be administered safely for prolonged periods, with appropriate monitoring. However, they are accompanied by drug-related side-effects [4,5]. Moreover, delivery of drugs to the joint by the oral route may not be efficient [6]. An alternative route is via intra-articular (IA) injection. IA steroidal anti-inflammatory drugs have been known to be effective against OA pain for decades, but IA administration has a limited duration of action with a maximum duration of eight weeks [7]. Additional drawbacks of IA injections include the need for re-injection, the risk of septic arthritis or systemic side effects [8,9]. Moreover, negative effects of IA corticosteroid injections on articular cartilage have been described in humans, horses and dogs [10,11]. Sustained release formulations which lower the frequency of re-injections and have decreased (systemic) peak levels of corticosteroid formulations would possibly mediate these setbacks [12]. Microspheres in drug delivery offer several advantages: encapsulated drugs are protected from degradation and/or clearance and the release kinetics of drugs can be adjusted [13]. Moreover, they can be injected in small volumes through small needles [13,14]. However, a poly lactic-co-glycolic acid (PLGA)-based microsphere formulation releasing triamcinolone acetonide (TA) aiming to achieve pain reduction in OA patients was recently shown not to be superior to bolus administration in a phase III trial [15]. This particular PLGA-based microsphere formulation had been tested pre-clinically in an acute arthritis rat model and shown to decrease pain over the course of 42 days [16].

Lately, several in vitro and preclinical datasets were obtained with an emerging drug delivery platform based on biodegradable amino acid-based polyesteramide (PEA). The PEA polymer is based on alpha-amino acids, aliphatic dicarboxylic acids and aliphatic α-ω diols [17], which provide functional groups for further modification of the polymer, to adjust physicochemical and degradation properties. The polymer consists of di-amino monomers connected with a di-acid linker in a polycondensation reaction and degrades through surface erosion via enzymatic reactions, thereby allowing constant drug release [18]. In contrast to PLGA, PEA polymers do not result in acidification [19,20]. PEA microspheres have been successfully applied as controlled drug delivery system for intraretinal [21,22] and intradiscal injections [14,23,24].

In the joint, PEA microspheres (PEAMs) showed retention of a loaded fluorescent label in the femorotibial joint of rats for up to 70 days after a single IA injection [12]. When loaded with TA, after an initial burst release, a gradual in vitro release over a period of six months was found [21]. In contrast, TA-loaded PLGA microspheres showed a stronger initial burst, a secondary burst and in-between periods of very limited drug release. Indeed, loaded with TA, the PEA platform showed up to three times longer analgesia and anti-inflammatory action compared to PLGA in an acute arthritis rat model [25]. In rodent models of OA, conflicting data were obtained for local TA release. In a chemically induced OA model, synovial inflammation was decreased in OA joints injected with TA-PEAMs after seven weeks, compared to empty PEAMs. Empty nor TA-PEAMs influenced articular cartilage quality [12]. However, in a rat model of instability-induced OA, local microspheres-based release of TA was shown to aggravate disease, which was not seen in the animals treated with bolus injection nor in healthy joints injected with TA-loaded PEAMs, indicating that the prolonged exposure to TA in combination with joint trauma was the cause [26]. Moreover, TA inhibited cell outgrowth from tissue, indicating a possible effect on tissue healing [26].

These preclinical data underline the importance and limitations of disease models used in OA. In experimental settings, OA is induced mostly by surgical, chemical or immunization techniques, as the moment of onset and disease progression can thereby be controlled. However, the initiating events and subsequent pathological changes are not directly comparable to spontaneous OA [27,28] and animal models are not always capable of correctly predicting clinical analgesic efficiency in human OA patients [29]. As opposed to rodent models, in dogs OA is a common chronic ailment, with 20% of adult dogs and 80% of geriatric dogs suffering from spontaneous OA [30,31]. In addition, there is great similarity between man and dog regarding joint anatomy and pathophysiology, including biomarkers [29,32,33]. Therefore, there is increasing attention for the use of companion dogs with spontaneous degenerative joint disease to clinically study the effect of treatments for OA [27,34,35,36], as part of the One Medicine vision [35]. Until now, only in healthy canines extended release corticosteroids were tested, showing cartilage degeneration that apparently recovered within half a year after injection [37]. The response to intra-articular application of corticosteroids in joints of canine patients with OA is still unknown.

The aim of this study was to assess the effects of continuous TA exposure on tissue regeneration by canine chondrocytes in vitro and for the first time compare this to intermittent exposure mimicking multiple IA injections in clinical practice. Subsequently proof-of-concept and safety were assessed of a single injection of TA-PEAMs in joints with spontaneous moderate to severe OA in a cohort of client-owned dogs, hereby taking the first step from rodent models to a large animal model of the PEA-TA formulation. It was hypothesized that a single IA injection would improve lameness and decrease the synovial PGE_2_ concentration, without significant adverse effects.

## 2. Materials and Methods

### 2.1. In Vitro Controlled Release of TA in Articular Chondrocyte Culture

Articular cartilage was harvested post mortem from the weight-bearing surfaces of femorotibial joints from healthy dogs sacrificed in unrelated experiments (approved by the Utrecht University Experimental Animal Ethics Committee, approval numbers #2016.II.529.002 and #2014.II.06.048). Chondrocytes were isolated by a 45 min enzymatic digestion in 0.15% *w*/*v* pronase (10,165,921,001, Roche Diagnostics, IN, USA) and 0.15% *w*/*v* collagenase overnight (LS004177, Worthington, Lakewood, NJ, USA) at 37 °C. Undigested debris was removed using a 70 μm cell strainer (352350, BD Biosciences, Franklin Lakes, NJ, USA) and cells where isolated upon washing with PBS. Chondrocytes were expanded in hgDMEM+Glutamax (31,966, Gibco Life Technologies, Bleiswijk, The Netherlands) containing 10% *v*/*v* FBS (16000–044, Gibco Life Technologies), 1% *v*/*v* penicillin/streptomycin (P11–010, PAA laboratories GmBH, Piscataway, NJ, USA), 0.1 mM ascorbic acid 2-phosphate (A8960, Sigma-Aldrich, Saint Louis, MO, USA), 10^−9^ M dexamethasone (D1756, Sigma-Aldrich), 1 ng/mL basic fibroblast growth factor (PHP105, AbD Serotec, Oxford, UK) and 0.05% *v*/*v* fungizone (15290–018, Invitrogen, Paisley, UK) at 37 °C, 21% O_2_ and 5% CO_2_. The culture medium was renewed every 3–4 days. At passage two, cells were cryopreserved in aliquots of 10^6^ cells per vial in hgDMEM+Glutamax with 10% *v*/*v* DMSO (20–139, Merck Millipore Corporation, Schiphol-Rijk, The Netherlands) and 10% *v*/*v* FBS, as per methods described previously [12,38]. Cells were thawed, expanded and passaged once before the experiment. First, cells were seeded onto a 96-well round bottom Ultra-Low Attachment Microplate (#7007, Corning^®^, Glendale, AZ, USA), at a density of 200,000 cells per well. Aggregates were formed by centrifugation of the 96-well plates at 300× *g* for 5 min. Aggregates were cultured in 200 μL medium DMEM supplemented with 2% insulin–transferrin–selenium (ITS)-X (51500056, Invitrogen), 2% ascorbate-2-phosphate (A8960, Sigma-Aldrich), 2% human serum albumin (HSA; Sanquin Blood Supply Foundation Amsterdam, The Netherlands), 1% penicillin/streptomycin (100 U/mL / 100 µg/mL). The following day, the medium was renewed before starting the experiment. Additionally, 0.1 μM TA (#T6501-250MG, Sigma-Aldrich) dissolved in 100% ethanol was administered directly to the medium on a daily basis during the 14-day culture experiment (“continuous exposure” mimicking continuous controlled drug delivery) at day 1–3 and day 11–13 and at day 1 and 8 (“intermittent exposure” mimicking clinical practice with repetitive IA injections). Each condition was analysed in six replicates and experiments were performed for two different donors. As a positive control for the chondrogenic potential of the chondrocytes, 10 ng/mL TGF-β1 (240-B, R&D Systems, Inc., Minneapolis, MN, USA) was used. Media were renewed twice weekly and stored at −20 °C for analysis of glycosaminoglycan (GAG) and DNA content. At day 14, pellets were digested overnight at 60 °C in papain (250 μg/mL papain (P3125, Sigma-Aldrich) + 1.57 mg cysteine HCL (C7880, Sigma-Aldrich). The 1,9-dimethylmethylene blue (DMMB) assay was used to quantify the GAG content of the pellets and media [39]. GAG concentrations were calculated by using chondroitin sulphate from shark cartilage (C4384, Sigma-Aldrich) as a standard, the absorbance was read at 540/595 nm. A PicoGreen assay (p11496, Life Technologies, Carlsbad, CA, USA) was used to determine the DNA content of the aggregates against a λ-DNA standard curve. Fluorescence was measured in a POLARstar Optima fluorescence microplate reader (Isogen Life Science, Utrecht, The Netherlands) at 485 nm excitation and 530 nm emission. After 14 days of culture, one aggregate per condition was fixed for 24 h in 4% neutral buffered formaldehyde (115935, Merck Millipore, Amsterdam, The Netherlands). The next day, the aggregates were embedded in paraffin and 5 μm sections were stained with Safranin O/Fast Green as described previously [40].

### 2.2. Preparation and Characterisation of Microspheres for In Vivo Application

**Figure 1:**
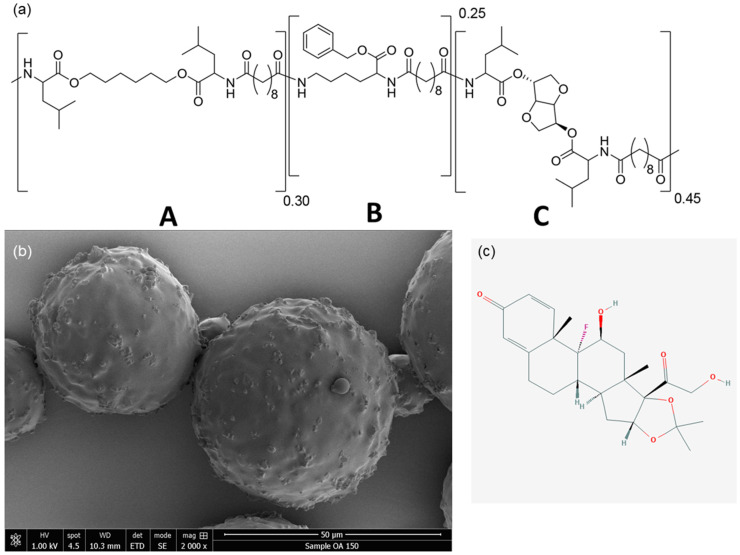
(**a**) Structure of polyesteramide (PEA) III Ac Bz random copolymer consisting of building blocks A, B and C (Leu, Lys, Leu). (**b**) Scanning electron microscopy (SEM) image of triamcinolone acetonide (TA) loaded microspheres. (**c**) 2D molecular formula of triamcinolone acetonide (TA) (https://pubchem.ncbi.nlm.nih.gov/image/imgsrv.fcgi?cid=6436&t=l, accessed date 31 January 2021).

Polyesteramide polymer (Figure 1a) and PEA-loaded microspheres (Figure 1b) were synthesized according to previously reported protocols [14,24,41] and were also used in a study on effectivity in a rat model [12]. Briefly, the polymer was prepared via solution polycondensation of di-p-tolue-nesulfonic acid salts of bis-(α-amino acid) α,ω-diol diesters, lysine benzyl ester and di-N-hydroxysuccinimide sebacate in anhydrous DMSO. The polymer was isolated from the reaction mixture in two precipitation steps. 1H nuclear magnetic resonance (NMR) spectra were obtained on a Bruker Avance 500 MHz Ultrashield NMR (Bruker Corporation, Billeria, MA, USA); samples were recorded in DMSO d6 (Sigma-Aldrich). Molecular weight and molecular weight distributions of PEA were determined by gel permeation chromatography equipped with a refractive index detector. Samples were dissolved in tetrahydrofuran at a concentration around 5 mg/mL and run at a flow rate of 1 mL/min at 50 °C. The molecular weights were calibrated along a narrow polystyrene standard calibration curve, using Waters Empower software (Waters Corporation, Milford, MA, USA) [12,24].

For the preparation of TA-loaded microspheres, PEA was dissolved in dichloromethane (Merck Millipore). 20 wt% TA (TEVA Pharmaceutical Industries, Rho, Italy) (Figure 1c) was added to the solution, which was homogenized by ultrasound. The suspension was added to 20 mL of an aqueous solution containing surfactants for stabilization (1 wt% of poly(vinyl alcohol and 2.5 wt% NaCl, (Sigma-Aldrich) under high shear, using an ultra-Turrax (IKA, Staufen, Germany). After a stable suspension was obtained the particles were allowed to harden in 100 mL of water containing 1 wt% of poly(vinyl alcohol) and 2.5 wt% NaCl for 12 h. Excess of water and surfactant was removed by rinsing and centrifugation. Finally, particles were frozen, dried and weighed in individual HPLC vials to the approximate amount of 40 mg PEAMs and γ-sterilized on dry ice. The size distribution of TA-loaded particles was determined with Static Light scattering, using a Malvern Mastersizer 2000 (Malvern Pananalytical, Malvern, UK) [12].

### 2.3. Release Kinetics of TA-Loaded Polyesteramide Microspheres in PBS

The microspheres used in the current study have been used and characterized in another study published recently [25]. Release of TA from PEA microspheres in PBS buffer was determined as described before [41]. Briefly, samples were incubated in a volume of 50 mL at 37 °C, of which 45 mL buffer was renewed. Buffer exchange was performed twice the first day, every day up to day 3 and from there every 3–4 days up to day 24. After that, the buffer was renewed on a weekly basis until day 70. The samples were analyzed for TA content by High-Performance Liquid chromatography (HPLC), using a Waters e2695 Alliance HPLC with UV detector (Waters Corporation) [25,41].

### 2.4. Veterinary Pilot Study Design

**Figure 2:**
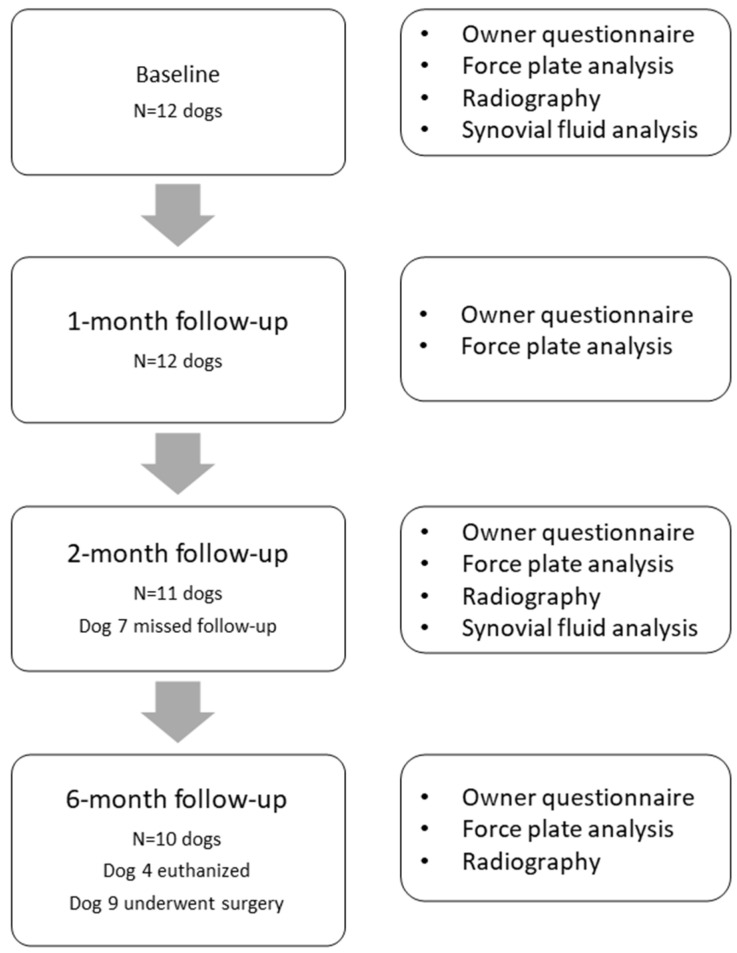
Schematic overview of the veterinary safety and proof-of-concept study.

This study was conducted with the approval of the Ethical Committee of the Department of Clinical Sciences, Utrecht University (#17-06). Owners were informed orally and in writing, and written consent was obtained before study enrolment. The study design is illustrated in Figure 2. Dogs were considered eligible for the study if they were otherwise healthy, weighed at least 15 kg, had a history of chronic lameness (lasting more than four weeks) attributable to a specific joint and were diagnosed with pain and OA of that particular joint on orthopaedic examination and radiographic evaluation. Dogs were excluded if they were gravid, had undergone surgery in the affected or contralateral limb in the preceding three months, if there was evidence of a fracture or neoplasia in the affected limb, or if the dog had received IA injections in the affected joint in the preceding three months. Dogs with joint instability on physical examination were also excluded from the study. Pain medication was discontinued four days prior to the start of the study, to obtain baseline levels of the read-out parameters and four days prior to each control visit to minimize the effect on read-out parameters.

After baseline clinical and kinetic evaluation (BM, AT), dogs were sedated, and the affected joint(s) were clipped and prepared aseptically. Arthrocentesis was performed, and synovial fluid (SF) was collected for cytology and stored at −20 °C. This was followed by IA administration of the TA-PEAMs through the same needle. Directly prior to injection, PEAMs were re-suspended in 2 mL (20 mg/mL) sterile 2% lidocaine HCl injection solution (B. Braun Medical, Melsungen, Germany), to avoid pain from the arthrocentesis procedure. Dogs with a body weight of 15–30 kg received 0.5 mL TA-PEAM solution (10 mg TA), dogs weighing 30–45 kg received 1 mL (20 mg TA) and dogs weighing over 45 kg received 1.5 mL (30 mg TA). Physical activity was limited to leash walks on the first two days after treatment. Thereafter, owners could gradually increase activity to the level before the start of the study. In case there was insufficient control of OA pain, relief analgesia in the form of NSAIDs or other analgesics was permitted during the study period, starting from three weeks after the IA injection. In such cases, owners were allowed to use the analgesics their pets were receiving prior to inclusion to the study. The dogs were evaluated after 1 and 2 months, and 6 months after IA injection (BM, AT).

#### 2.4.1. Physical Examination and Lameness Score

All dogs underwent a full physical (including orthopaedic and neurologic) examination by a board-certified veterinary surgeon (BM). Lameness was recorded on a 4-point scale, ranging from 0 (none), 1 (intermittent mild lameness after rest and exercise), 2 (mild lameness/intermittent moderate lameness after rest and exercise), 3 (moderate lameness/non-weight-bearing after exercise) or 4 (non-weight-bearing lameness) [42] (BM, AT).

#### 2.4.2. Kinetic Gait Analysis

Ground reaction forces (GRFs) were measured by force plate analysis (FPA) with a quartz crystal piezoelectric force plate (Kistler type 9261, Charnwood Dynamics Limited, Rothley, UK) together with the Kistler 9865E charge amplifiers as described previously [43,44]. Data recorded were considered valid when in the same run the thoracic limb followed by the ipsilateral pelvic limb contacted the force plate completely. Each pass across the plate was evaluated by an observer to confirm foot strikes and walking gait. Trials were discarded for distracting head motions or irregularities in the gait. A minimum of 8 recordings per thoracic and per pelvic limb were used for data processing. GRFs in the mediolateral (Fx), craniocaudal (Fy) and vertical (Fz) direction were normalized for body weight (N/kg). Symmetry indices (SI) were calculated according the following formula:

(1) $$\frac{\left(\mathrm{contralateral}\;\mathrm{limb}\;\mathrm{GRF}\;-\;\mathrm{affected}\;\mathrm{limb}\;\mathrm{GRF}\right)}{\left(0.5\;*\left(\mathrm{contralateral}\;\mathrm{limb}\;\mathrm{GRF}\;+\;\mathrm{affected}\;\mathrm{limb}\;\mathrm{GRF}\right)\right)}*100$$

SIs were determined for peak propulsive force (PPF), peak vertical force (PVF) and area under the force–time curve of Fz+, which is equal to the vertical impulse (VI) during the stance phase, as previously described [45,46]. The affected joint was defined as the joint that at the start of the study was considered painful and was treated IA with TA-PEAMs.

#### 2.4.3. Owner Assessment of Pain and Lameness

A questionnaire to owners regarding pain-related behaviour and function of their dog was used to assess the owners’ perspective of treatment outcome (Table 1) and was partially adapted from the Canine Brief Pain Inventory [47] and supplemented with questions relating to mobility of the dog. To accommodate the owners, the scales were flipped: a score of 1 indicated the worst score, and 10—an excellent score. Relief analgesia in case of recurrence or persistence of clinical signs was recorded by the owners.

#### 2.4.4. Radiographic Evaluation

**Figure 3:**
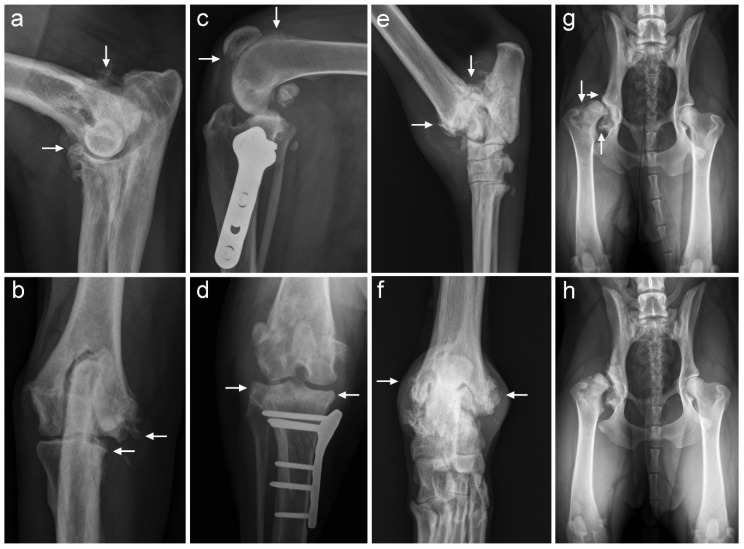
Representative examples of radiographs obtained 6 months after intra-articular injection with triamcinolone acetonide-loaded polyesteramide microspheres. Arrows indicate locations for osteophyte measurements. (**a**,**b**) Mediolateral and craniocaudal radiographs of the elbow joint of dog 6. (**c**,**d**) Mediolateral and caudocranial radiograph of the knee joint of dog 7. (**e**,**f**) Mediolateral and plantarodorsal radiographs of the tarsal joint of dog 5. (**g**,**h**) Ventrodorsal radiograph of the pelvis of dog 3 prior to and 6 months after treatment, respectively.

Orthogonal radiographs of the affected joints were obtained within one month prior to inclusion in the study and at two and six months after injection to monitor for adverse effects and OA progression. Images were examined blinded, in one session, by a board-certified veterinary radiologist (MB). Each radiograph was scored for OA severity as follows: 0 (none); 1 (mild), 2 (moderate) or 3 (severe) [48]. To assess OA more quantitatively, the size of osteophytes was measured and graded as described previously for the elbow joint: 0 (no OA), 1 (osteophytes < 2 mm), 2 (osteophytes 2–5 mm) or 3 (>5 mm) [49]. Osteophytes in cubital joints were measured at the cranial aspect of the radial head, the caudal surface of the lateral condylar ridge, the medial contour of the humeral condyle and the medial contour of the medial coronoid process. For coxofemoral joints, osteophytes on the edge of the cranial and caudal acetabulum or at the femoral neck were considered. For femorotibial joints, osteophytes were measured at the proximal trochlear edge, the distal patella and the lateral and medial tibia plateau. Osteophytes in the tarsal joint were measured at the medial and lateral aspect of the distal tibia (Figure 3a–h). The highest value for each joint was considered in the analysis.

#### 2.4.5. Synovial Fluid Analysis

Before treatment and two months after IA injection, arthrocentesis was performed to collect SF. Direct impression smears were stained with Hemacolor^®^ and assessed for number of cells, the type of cells (synoviocytes, macrophages, polymorphonuclear leukocytes, lymphocytes), the presence of cell clusters and the presence of microorganisms. Cells were counted with an automatic cell counter (1450102, Bio-Rad, Hercules, CA, USA) using trypan blue dye. Any remaining SF was aliquoted per 100 µL to avoid excessive freeze–thaw cycles and stored at −20 °C for biochemical analysis. GAG concentration was determined as described previously [39]. In short, after incubation with 0.01 mg/mL hyaluronidase (H2126, Sigma-Aldrich) at 37 °C for 30 min, GAG concentration of the SF (diluted 1:50 with PBS-EDTA) was determined by the DMMB assay [39] using chondroitin sulphate from shark cartilage (C4384, Sigma-Aldrich) as a standard. The absorbance was read at 540/595 nm (Multimode detector DTX 880, Beckman Coulter, Brea, CA, USA). Prostaglandin E_2_ (PGE_2_) levels were determined by ELISA (1:10 diluted in assay buffer, 514010, Cayman Chemical, Ann Arbor, MI, USA) following manufacturer’s instructions. Samples were analysed in triplicates and were analysed all at the same time. To aid in the interpretation of SF biomarker levels, SF of macroscopically healthy joints collected post mortem from six experimental dogs in unrelated experiments (approved by the Dutch Central Committee for Animal experimentation, experimental number: #AVD108002015282, approval date 25/11/2015) and six client-owned dogs with OA presented for orthopaedic joint surgery were analysed concurrently.

### 2.5. Statistical Analysis

Statistical software (IBM SPSS Statistics 24, Armonk, NY, USA) was used for all comparisons. Normality of the data was checked by assessing the Q–Q plots, histograms and Shapiro–Wilk tests. A one-way ANOVA was used to analyse GAG/aggregate, DNA/aggregate and GAG production. A repeated measures ANOVA was used to assess differences in GAG release in the in vitro study and the body weight, questionnaire scores, and force plate parameters at baseline, 1, 2 and 6 months of the in vivo study. PGE_2_ and GAGs SF levels at baseline and 2 months were compared using a Mann–Whitney U test. The visual lameness scores obtained at baseline, 1, 2 and 6 months were compared using the Wilcoxon’s signed rank test, as were the OA and osteophyte scores at baseline, 2 and 6 months. *p*-value < 0.05 were considered statistically significant after correction for multiple testing (Benjamini–Hochberg method).

## 3. Results

### 3.1. Characterization of PEA Microspheres and Release of TA from PEA Microspheres in PBS

The microspheres used in the current study have been used and characterized in another study published recently [25]. The average size of the microparticles was 22.4 μm (ranging from 8 to 50 μm), with a polydispersity index of 1.205. Loading with TA was 20 wt%, with 12% located outside of the spheres. SEM analysis of the TA-loaded microspheres is shown in Figure 1b. TA release in PBS buffer at 37 °C for 24 weeks, as previously published [25], showed an initial burst at day one with a further gradual increase of TA release up to 60% cumulative release after 175 days [25].

### 3.2. In Vitro Effect of TA at Different Treatment Regimes in Articular Chondrocyte Culture

**Figure 4:**
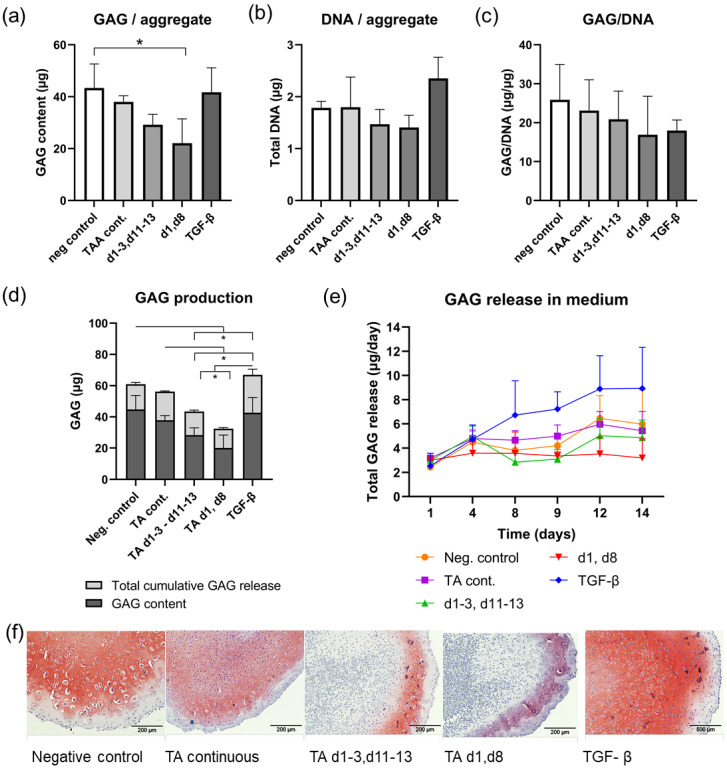
Biochemical and histological analysis of aggregates exposed to continuous or intermittent TA. (**a**) Total GAG per aggregate was significantly lower in the condition that received TA on day 1 and 8 compared to the negative control of the 14-day experiment. (**b**) DNA per aggregate and (**c**) GAG/DNA did not differ between culture conditions after the 14-day culture experiment. (**d**) Total GAG production and GAG release and (**e**) GAG release in the culture medium showed a similar pattern, in which total GAG production and release were significantly lower in the conditions that received TA intermittently (TA d1–3, d11–13 and TA d1,d8) compared to the other conditions. (**f**) Safranin-O/Fast Green staining of representative aggregate paraffin sections. * indicates significant difference (*p* < 0.05).

After 14 days of culture, total GAG content of the chondrocyte aggregates exposed to TA intermittently at day 1 and day 8, but not during d1–3 and d11–13, was significantly lower than in untreated aggregates (*p* = 0.038; Figure 4a). Release of GAGs in the culture medium and total GAG production (GAG content + release) showed a similar pattern as the GAG content of the aggregate (Figure 4d,e). Total GAG production was lower in the aggregates exposed to TA at day 1 and day 8 and during d1–3 and d11–13, compared to the negative and positive (TGF-β) and continuous TA exposure (Figure 4d). GAG release in the culture medium was not significantly different between conditions after correction for multiple testing, nor was the GAG/DNA (Figure 4b) and DNA content (Figure 4c). Safranin-O/Fast Green staining showed decreased Safranin-O staining in the two conditions with intermittent TA exposure (Figure 4f).

### 3.3. Veterinary Pilot Study

#### 3.3.1. Study Population and Visual Lameness Score

Seventeen dogs were screened for inclusion of the study (Figure 2). Twelve dogs met the inclusion criteria and were enrolled in the study and suffered from symptomatic OA of the cubital, coxofemoral, femorotibial or tarsal joint (Supplementary Table S1). Two dogs received the IA injection with TA-PEAMs in two joints: dog 3 (right coxofemoral and right femorotibial joint) and dog 5 (right cubital joint and left tarsal joint). The median age at inclusion was 8 years (range, 1–12 years) and the average body weight 30 kg (range, 21–36 kg). Body weight did not change during the course of the study (*p* = 0.74). The visual lameness scores included mild (2/4; *n* = 7), moderate (3/4; *n* = 5) and non-weight-bearing (4/4; *n* = 1) lameness (Supplementary Table S2). Physical and radiological findings were compatible with OA. No serious adverse events were reported by the owners after IA TA-PEAMs injection. Transient polyuria (circa 1 week) was reported in 5/12 dogs (42%).

**Figure 5:**
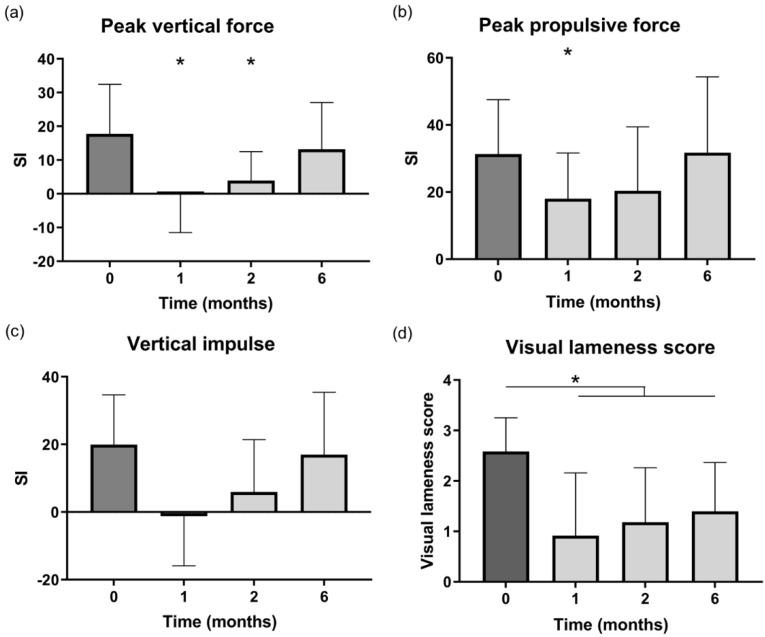
Results of force plate analysis before and 1, 2 and 6 months after intra-articular injection with triamcinolone acetonide-loaded microspheres. (**a**) The symmetry index (SI) of the peak vertical force (**a**) was improved at one and two months post injection (*p* = 0.04, *p* = 0.027) compared to baseline. (**b**) The peak propulsive force was improved at the one-month follow-up (*p* = 0.041). (**c**) There was a borderline significant decrease in vertical impulse SI at one and two months post treatment (*p* = 0.053, *p* = 0.062). (**d**) The visual lameness score was significantly lower compared to baseline during the entire study period. * Indicates significantly different from baseline (*p* < 0.05).

Visual lameness scores significantly improved at one month (*p* = 0.031), two months (*p* = 0.011) and six months (*p* = 0.016) after IA injection of TA-PEAMs (Figure 5d, Supplementary Table S2).

Three months after inclusion in the study dog 4 remained lame, and additional CT images were obtained in which a large fragmented coronoid process (FCP) was found. Dog 4 underwent arthrotomy to remove the large FCP, clinical signs unfortunately did not improve after surgery and the dog was eventually euthanized two months thereafter. Dog 9 was lost for the six months follow-up, since a proximal abducting ulnar osteotomy in the contralateral limb was performed five months after IA TA-PEAMs injection.

During the two-month follow-up period, 1 out of 12 owners provided their dogs with additional pain relief medication consisting of an opioid drug (Tramadol). In the following period up to six months after the IA TA-PEAMs injection, 3 out of 10 owners reported administration of additional (relief) analgesics (Supplementary Table S1).

#### 3.3.2. Kinetic Gait Analysis

Force plate analysis was performed in dogs prior to treatment and after one month, and after two and six months (Figure 5a–c). The symmetry indices of the PVF (Figure 5a) and PPF (Figure 5b) were significantly improved from 18 to 0 and 31 to 17, respectively, at one month post injection (*p* = 0.04, *p* = 0.041) and PVF remained improved at the two-month follow-up (*p* = 0.027). There was a borderline significant decrease in VI asymmetry (Figure 5c) at one and two months post treatment (*p* = 0.053, *p* = 0.062). Six months after IA injection, none of the force plate parameters did significantly differ from baseline values.

#### 3.3.3. Radiographic Evaluation

The severity of OA and osteophyte size were evaluated in the subset of patients that completed the follow-up period, at two and six months after IA administration of TA-PEAMs and compared with pre-treatment radiographs. No significant changes in OA severity or osteophyte size were detected in any of the dogs (*p* > 0.1) (Supplementary Table S3).

#### 3.3.4. Owner Assessment of Pain and Lameness

Questionnaires were completed by owners prior to treatment, and 1, 2 and 6 months after IA injection (Table 1). There was a significant improvement in total questionnaire scores) after 1 (*p* = 0.0008), 2 (*p* = 0.023), and 6 months (*p* = 0.018) compared to baseline.

#### 3.3.5. Synovial Fluid Analysis

**Figure 6:**
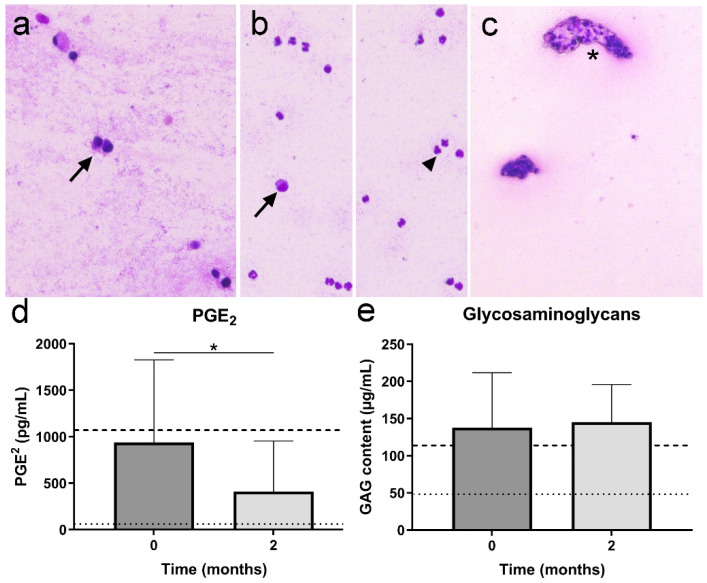
Results of the synovial fluid (SF) analysis prior to (T = 0) and at two-month follow up upon intra-articular injection with triamcinolone acetonide-loaded microspheres. (**a**) Synovial aspirate of dog five, six months after treatment. A few synoviocyte-like cells are present (arrow). (**b**) Cytological smear of SF of dog 3, six months after treatment. Abundant polymorphonuclear cells are present (arrowhead), along with some macrophage-like cells (arrow). No (intra)cellular bacteria were found. (**c**) Contact smear of the SF of dog 1 prior to treatment. Clusters of synovial lining cells are present (asterisk). Concentrations of (**d**) prostaglandin E_2_ (PGE_2_), (**e**) glycosaminoglycans (GAGs) in SF before and two months after intra-articular injection with triamcinolone-acetonide-loaded microspheres. Dotted lines indicate SF control samples of six healthy joints, dashed lines indicate average SF biomarker values for osteoarthritic joints of 6 donors. * Indicates significant difference (*p* < 0.05).

Prior to IA TA-PEAMs injection, SF from 9 out of the 12 joints included was available for analysis. Cytology confirmed the presence of OA (9/9), showed an increased number of synoviocytes and macrophage-like cells (8/9; Figure 6a,b), and the presence of cellular clusters (4/9; Figure 6c). In dog 8 there was also an abundance of polymorphonuclear leukocytes (Figure 6b). No (intracellular) bacteria were observed on cytology or culture excluding joint sepsis. The mean total cell count was 6.3 × 10^6^ (range 1.3–10.4 × 10^6^) cells/mL. At the two-month follow-up visit, seven samples were available for analysis and in one put of seven samples, an increase of polymorphonuclear leukocytes was noted (knee joint, dog 3). No (intracellular) bacteria were observed on cytology or culture excluding joint sepsis. The average total cell count was 4.3 × 10^6^ cells/mL (range 1.7–9.9 × 10^6^) and was not significantly different from baseline (*p* = 0.34). PGE_2_ was significantly lower two months after IA TA-PEAMs injection, compared to pre-treatment values (*p* = 0.045; Figure 6d). No significant changes in total GAG content in the SF were detected two months after IA TA-PEAMs injection compared to baseline measurements (*p* = 0.345; Figure 6e).

## 4. Discussion

The goal of this study was to investigate safety and preliminary efficacy of controlled release of TA in canine spontaneous OA, as a more representative model for OA therapy in man. Since conflicting data were obtained previously on the effect of corticosteroids on (canine) cartilage, first the effect was determined of the continuous presence of TA on canine chondrocyte-mediated tissue regeneration, mimicking the long-term presence of TA by controlled release. TA was added directly to the culture medium to compare the intermittent and continuous exposure of TA to chondrocytes, as biocompatibility of PEA microspheres and short-term effects of release of TA in chondrocyte culture were shown before [12]. No inhibitory effect was noted, whereas surprisingly, short intermittent exposure inhibited proteoglycan production by the chondrocytes. Although the proteoglycan decrease was not significant with the longer intermittent exposure periods, an inverse relation between length of exposure period and proteoglycan content was apparent. The mechanism behind this phenomenon is unclear. Similar differences between effects of intermittent and continuous exposure have been described before in chondrocyte culture. Intermittent exposure to PTH enhanced osteogenic differentiation of rat condylar chondrocytes, whereas continuous exposure maintained their cartilage phenotype [50]. Additionally, intermittent vitamin D exposure resulted in higher growth plate chondrocyte proliferation than its continuous presence, which appeared to be related to a decrease in vitamin D receptor expression in the latter condition [51]. Moreover, a growth factor recently shown to have a structure-modifying effect in OA clinical trial sprifermin (FGF18), was also more effective in enhancing matrix production by porcine articular chondrocytes upon intermittent than continuous exposure [52]. As corticosteroids are known to downregulate the expression of their own receptors [53], this could explain the effect observed in the current study. This suggests corticosteroid signalling has an intrinsically negative effect which is mitigated by lasting receptor downregulation upon long-term presence of their ligands, and hence, long-term exposure may not affect cartilage matrix synthesis in vivo. To what extent the intermittent exposure can be viewed as fully mimicking repeated injection of TA in clinical practice may be a matter of debate, as generally repeated injections are not performed within the short time span of this in vitro study. Studies using articular cartilage explants rather than chondrocyte culture may allow for longer culture periods to verify this aspect [54]. No significant increase in GAG and DNA levels was noted in the chondrocyte aggregates stimulated with TGF-β, compared to the basal culture conditions. Why TGF-β did not enhance GAG production is unclear, but it could be due to insensitivity of canine articular chondrocytes to TGF- β as the sole chondrogenic stimulant, as was found recently in canine adipose tissue-derived mesenchymal stem cells, or due to environmental factors during culture [55].

Upon administration of TA-loaded PEAMs in a proof-of-concept study of canine patients with severe OA, most animals improved clinically, according to objective and subjective gait analyses and the owner questionnaire. Clinical improvement was most apparent during the first two months. The use of canine test subjects in studies evaluating analgesic treatment poses some obvious difficulties, which partially can be overcome by a combination of patient-driven outcomes (e.g., owner questionnaires) in combination with objective kinetic gait analysis [45,56]. With respect to the latter, Fanchon et al. proposed cut-off values for the PVF for non-lame dogs: <3.5% asymmetry, with 85% sensitivity and 80% specificity [57]. When considering the results of the present study in this light, all dogs showed asymmetry prior to treatment, which decreased to 3/12 and 5/11 of the dogs one and two months after treatment. Six months after IA treatment with TA-loaded PEAMs, significant improvement was still appreciated in six out of ten owner questionnaires but not on force plate analysis, with two out of ten dogs showing no lameness based on the aforementioned rule. A discrepancy between owner assessment reflected in the questionnaire and force plate analysis could be explained by the fact that the owners were also focused on other behaviours than lameness (e.g., the ability to perform its daily activities, such as going for a walk), when making efficacy evaluations in their dogs [47]. Furthermore, force plate measurements reflect a single moment in time, whereas the owners monitor their dogs’ behaviour on a daily basis. With the particular study design in which a cohort of patients is IA treated with TA-PEAMs, confirmation bias could also play a role in the assessing owners (and veterinarians). This preliminary study (analogous to a phase I/IIa study in human patients) was undertaken in a small cohort of animals with severe OA without alternative treatment options first, to primarily test safety, given the aforementioned conflicting results of long-term IA corticosteroid exposure in rat models. Altogether, in the light of the relatively small study population, intended to study safety and provide some proof of concept, the severity of disease and the heterogeneity in terms of affected joints and previous treatments, a more standardized and placebo-controlled canine OA patient study will be the next step towards confirming the efficacy of pain relief in objective parameters for six months, which would be the first OA treatment to achieve this.

Joint health was monitored with the aid of biomarkers indicative for inflammation and cartilage degradation. The pro-inflammatory mediator PGE_2_ is known to be increased in human patients with symptomatic OA [58], and also directly influences nociceptors, mediating OA pain [59]. In dogs with experimentally induced OA, PGE_2_ concentrations in SF correlated well with force plate analysis and subjective lameness scores [60], providing evidence for the use of PGE_2_ as a valuable biomarker in the evaluation of canine OA. Indeed, in this study, total SF PGE_2_ levels tended to decrease after IA TA exposure, consistent with improvement in clinical lameness. Several studies showed an increase in (fragments of) GAGs in the SF of OA joints, compared to healthy joints in dogs [61,62] and horses [63,64], reflecting the increased activity of proteases in OA cartilage, leading to cartilage matrix loss. In the SF samples in the present study, SF GAG levels remained unchanged in the two months after IA injection, which could indicate no (long-term) change in cartilage breakdown further supporting the inferred safety of continuous TA exposure shown in vitro in the present study and in vivo upon the IA TA-PEAM treatment. In order to minimize the influence of additional pain medication on the synovial PGE_2_ content at the start of the study, owners had to discontinue additional pain medication at least four days prior to control visits. Although owners were allowed to administer additional pain medication during the study, which could have affected PGE_2_ levels measured at two months post injection, this was done for one patient only. As in this case the pain medication concerned an opiate, this could not have affected the PGE_2_ measurement.

Clinical side effects were transient polydipsia and polyuria, which is commonly reported after corticosteroid administration in dogs [65]. Here this was most likely due to the initial TA ‘burst release’ reaching the systemic circulation from the synovial space, although a lower peak plasma level was demonstrated in rats after IA administration in PEAMs compared to a commercially available TA formulation (Kenalog^®^) [12]. However, no relationship was apparent between the occurrence of polyuria and body weight (reflecting the exact dosage of TA administered). Furthermore, since PEAM degradation mainly occurs through enzymatic degradation, degradation and thus drug release is accelerated in OA joints vs. healthy joints [66]. In joints with higher inflammation grades, degradation is expected to be faster through enhanced enzyme activity, and this could explain an enhanced burst release and subsequent polyuria/polydipsia in a subset of the patients. The present study entails a limited number of dogs and, unfortunately, pre-treatment SF PGE_2_ values were not available for all dogs to verify whether inflammation was indeed more pronounced in this subset of animals. Another side effect was transient polyphagia (a common side effect of corticosteroid administration in dogs) in fewer dogs, but otherwise no minor or major side effects were noted by the owner nor the veterinarian.

When extrapolating these results to continuous exposure of other corticosteroids, caution is required. There are different types of corticosteroid formulations and their biologic effects differ depending on the type used. A recent review on the use of IA corticosteroids concluded that the results of different corticosteroids should not be generalised, as methylprednisolone acetate consistently caused deleterious effects, while IA TA demonstrated favourable effects on clinical, synovial and cartilage parameters. Moreover, negative effects also seemed to be dose-dependent, as higher dosages of corticosteroids were associated with gross cartilage damage and chondrotoxicity [67]. It should be noted, however, that the in vivo effects of methylprednisolone acetate may actually at least partially be explained by the vehicle used. Most likely due to a high concentration of PEG, it was shown to induce tissue degeneration in intervertebral discs and the spinal cord [68]. For TA, in the horse both in vitro and in vivo experimental studies beneficial effects were found at low doses and durations [10,69]. Moreover, in human tissue, TA inhibited loss of proteoglycans in co-culture of human cartilage tissue with synovium [70], and in equine cartilage stimulated with IL-1β decreased COX-2 and matrix metalloproteinase gene expression [71]. However, prolonged intra-articular exposure of TA in joints with acute trauma must be avoided, as it has been found to exacerbate joint instability and associated joint degeneration in an experimental model [26].

In canines until now the majority of studies was performed in experimental animal models and little is published on IA corticosteroid therapy in spontaneous OA [11,72,73,74]. We found PEAM-mediated delivery of TA was safe with preliminary efficacy, with a promise for truly long-term pain relief. Re-injections would be required on a less frequent basis, limiting the well-known drawbacks of frequent reinjection of corticosteroids [75]. As the incidence of OA in canines is high, such treatment would not only fulfil an unmet need in the human clinic, but also in veterinary practice. However, given the exploratory approach without a control group, and heterogeneity of the study population, conclusions regarding clinical efficacy should be further substantiated in a randomised placebo-controlled study.

## 5. Conclusions

This proof-of-concept study showed that in vitro, continuous TA exposure appeared superior to intermittent TA exposure to articular canine chondrocytes. Safety and preliminary clinical efficacy of IA treatment with controlled release of TA from a biomaterial-based platform was shown in client-owned dogs with OA, as a model for the human patient. Large placebo-controlled studies need to be done to consolidate these findings.

## Figures and Tables

**Table 1:** Responses to owners’ questionnaires before and one, two and six months after intra-articular injection with triamcinolone acetonide-loaded microspheres.

Questions	Before Treatment (*n* = 12)	After 1 Month (*n* = 11)	After 2 Months (*n* = 12)	After 6 Months (*n* = 10)
Does your dog show lameness, and in which severity?	3.5 (2–10)	8 (1–10) *	7 (3–10)	5.5 (3–10)
Does your dog have pain as a result of its osteoarthritis?	4 (1–10)	8 (1–10) *	7 (1–10)	6 (3–10)
Does your dog show any weakness in its affected leg?	6.5 (1–10)	9.5 (3–10) *	9 (3–10)	8 (3–10)
Does your dog have difficulty rising up?	4 (1–10)	8 (2–10) *	8 (2–10)	6.5 (1–10)
Does your dog have difficulty lying down?	4.5 (1–10)	9 (4–10) *	9 (4–10) *	8.5 (1–10)
Does the pain interfere with normal activities in and around the house?	5.5 (2–10)	9 (5–10) *	9 (3–10)	8 (1–10)
Does the pain interfere with the quality of life of your dog?	4.0 (1–10)	8 (2–10) *	8 (2–10) *	8 (3–10) *
Does the pain interfere with the ability to walk?	3.5 (1–7)	6 (2–10) *	6 (1–10)	6 (2–10) *
Does the pain interfere with the quality to run?	3.5 (1–6)	7 (1–10) *	5 (1–10) *	5.5 (2–10) *
Does the pain interfere with the ability to walk stairs?	4.0 (1–10)	7 (1–10)	8 (1–10)	5.5 (1–10)

Data represented as median (range). * Indicates significant difference from baseline (*p* < 0.05). To accommodate the owners, a score of 1 indicated the worst score, and 10 indicated an excellent score.

## Data Availability

Data will be made available on a reasonable request.

## Supplementary Materials

The following are available online at https://www.mdpi.com/1999-4923/13/3/372/s1, Table S1: Overview of dogs included in the study; Table S2: Visual lameness score per dog per time point; Table S3: Blinded evaluation of severity of osteoarthritis and osteophyte size on radiographs before, and 2 and 6 months after intra-articular injection with triamcinolone acetonide-loaded microspheres.
