# Supplementary material for: Intra-Articular Slow-Release Triamcinolone Acetonide from Polyesteramide Microspheres as a Treatment for Osteoarthritis
Source: Pharmaceutics. 2021 Mar 11;13(3):372. doi: 10.3390/pharmaceutics13030372 (PMC7999265; doi:10.3390/pharmaceutics13030372)
Supplement: Supplementary file 1 [file pharmaceutics-13-00372-s001.pdf]

pharmaceutics

Supplementary Materials: Intra-Articular Slow-Release Triamcinolone Acetonide from Polyesteramide Microspheres as a Treatment for Osteoarthritis

Anna Tellegen [1], Martijn Beukers [1], Imke Rudnik-Jansen [2], Nicolien van Klaveren [3], Kan Loi How [4], Nina Woike [5], George Mihov [5], Jens Thies [5], Erik Teske [1], Laura Creemers [2], Marianna Tryfonidou [1,*] and Björn Meij [1]

Table S1: Overview of dogs included in the study.

Dog	Joint	Breed	Age (yrs)	Body weight (kg); (BCS)	Diagnosis	Treatment History	Additional Pain Relief Medication
1	R Hip	Rottweiler	1	35 (5/9)	HD, OA	Conservative (NSAIDs)	-
2	R elbow	Labrador retriever	3	32 (6/9)	OA	Arthrotomy 6 mo earlier	-
3	R knee, R hip	Bouvier de Flandres	9	36 (5/9)	OA	TPLO 10 mo earlier, MMX 4 mo earlier	9Wk PI q24h 20 mg methylprednisolone + q24h 300mg tramadol
4	L elbow	Labrador retriever	9	32 (7/9)	OA	Arthroscopy 3 yrs earlier	3Wk PI q8h 100mg Tramadol
5	R elbow, L tarsus	Cesky fousek	12	22 (5/9)	OA	Conservative, arthroscopy 6 yrs earlier	
6	R elbow	Labrador retriever	11	29 (6/9)	FCP, OA	Conservative	
7	R knee	Siberian husky	8	30 (5/9)	CCL rupture, OA	TPLO	-
8	R elbow	Shar Pei	4	33 (6/9)	FCP, MCD, OA	Arthrotomy 4 yrs earlier, arthroscopy 3 mo earlier	4Mo PI: 1 q24h 227mg firocoxib
9	R elbow	Belgian Shepherd dog	6	29 (7/9)	MCD, OA	Arthroscopy	-
10	R elbow	Australian cattle dog	8	21 (6/9)	FCP, MCD, OA	Arthrotomy 1 yr earlier,	8Wk PI q24h 100mg carporal
11	R elbow	German Shepherd dog	8	36 (5/9)	FCP, OA	Conservative	-
12	R hip	Wetterhoun	5	25 (6/9)	HD, OA	TPO 4 yrs earlier with implant removal 3 mo later due to implant infection	-

Abbreviations: yrs, years; BCS, body condition score; R, right; HD, hip dysplasia; OA, osteoarthritis; L, left; NSAIDs, non-steroidal anti-inflammatory drugs; mo, months; Wk, week; PI, post-injection; TPLO, tibial plateau levelling osteotomy; MMX, meniscectomy; FCP, fragmented coronoid process; MCD, medial compartment disease; CCL, cranial cruciate ligament; TPO, triple pelvic osteotomy.

Table S2: Visual lameness score per dog per time point.

Dog	Limb	Before Treatment	After 1 Month	After 2 Months	After 6 Months
**1**	RH	2	2	0	0
**2**	RF	2	2	1	0
**3**	RH	4	3	3	3
**4**	LF	3	3	3	N/A
**5**	RF	3	1	2	2
**5**	LH	3	1	2	2
**6**	RF	3	-1	1	2
**7**	RH	3	0	N/A	2
**8**	RF	2	-1	0	1
**9**	RF	3	-1	1	N/A
**10**	RF	2	0	1	2
**11**	RF	2	0	1	1
**12**	RH	2	0	0	1

A negative value indicates lameness of the contralateral limb. Abbreviations: LF, left front; LH, left hind, RF, right front; RH, right hind. N/A, not available.

Table S3: Blinded evaluation of severity of osteoarthritis and osteophyte size on radiographs before, and 2 and 6 months after intra-articular injection with triamcinolone acetonide-loaded microspheres.

		Osteoarthritis Score			Osteophyte Size (largest, mm)		
**Dog**	**Joint**	**Before Treatment**	**After 2 Months**	**After 6 Months**	**Before treatment**	**After 2 Months**	**After 6 Months**
1	Hip	3	3	3	6.0	6.0	5.8
2	Elbow	2	2	2	3.7	4.4	4.5
3	Knee	3	3	3	4.9	4.8	3.9
3	Hip	3	3	3	4.4	3.5	5.2
4	Elbow	3	3	N/A	4.5	4.8	N/A
5	Elbow	3	3	3	7.2	7.1	7.5
5	Tarsus	3	3	3	5.0	4.9	5.6
6	Elbow	3	3	3	6.0	6.2	6.4
7	Knee	2	N/A	2	4.9	N/A	4.8
8	Elbow	2	2	2	3.4	3.7	3.7
9	Elbow	3	3	N/A	5.8	5.9	N/A
10	Elbow	3	3	3	5.5	5.8	5.8
11	Elbow	3	3	3	5.7	6.1	6.2
12	Hip	1	1	1	1.7	1.9	1.9

N/A, not available.
